# Local injection of high-molecular hyaluronan promotes wound healing in old rats by increasing angiogenesis

**DOI:** 10.18632/oncotarget.23246

**Published:** 2017-12-14

**Authors:** Luying Huang, Yi Wang, Hua Liu, Jianhua Huang

**Affiliations:** ^1^ First Affiliated Hospital of Jinzhou Medical University, Jinzhou, China; ^2^ School of Pharmaceutical Science, Jinzhou Medical University, Jinzhou, China; ^3^ Third Affiliated Hospital of Jinzhou Medical University, Jinzhou, China; ^4^ Graduated School of Jinzhou Medical University, Jinzhou, China

**Keywords:** high molecular hyaluronan, wound healing, angiogenesis, aging

## Abstract

Impaired angiogenesis contributes to delayed wound healing in aging. Hyaluronan (HA) has a close relationship with angiogenesis and wound healing. However, HA content decreases with age. In this study, we used high molecular weight HA (HMW-HA) (1650 kDa), and investigated its effects on wound healing in old rats by local injection. We found that HMW-HA significantly increases proliferation, migration and tube formation in endothelial cells, and protects endothelial cells against apoptosis. Local injection of HMW-HA promotes wound healing by increasing angiogenesis in old rats. HMW-HA increases the phosphorylation of Src, ERK and AKT, leading to increased angiogenesis, suggesting that local injection of HMW-HA promotes wound healing in elderly patients.

## INTRODUCTION

With aging population increasing worldwide, delayed wound healing in the elderly is a clinical challenge [1]. Wound repair is a multistep process consisting of hemostasis, inflammatory cell infiltration, tissue re-growth and remodeling [2]. In elderly population, this progression of events is altered, resulting in wounds that heal more slowly compared with wounds in young adults [3]. One of the most important factors leading to impaired wound healing in elderly patients is related to insufficient blood supply to the wound area. Because angiogenesis in the elderly is impaired [4, 5], new ways to increase angiogenesis is critical to promote wound healing.

HA is a polysaccharide belonging to the glycosaminoglycan family of macromolecules. This biopolymer consists of repeated disaccharides of (β,1–4)-D-glucuronic acid-(β,1–3)-*N*-acetyl-D-glucosamine [6]. HA is a ubiquitous component of tissue ECM but is found in particularly high concentrations as a native homeostatic form within the hydrated tissues such as the vitreous of the eye, articular cartilage, lymphatics and skin. HA plays an important role in maintaining tissue homeostasis, including angiogenesis. It has been reported that native high molecular-weight HA (HMW-HA) is anti-angiogenic [7], whereas degradation products that are of specific size (e.g., 3–10 disaccharide units; o-HA) exhibit pro-angiogenic activity [8, 9]. However, native HA is degraded into millions of different-sized fragments as a result of the enzymatic activity of hyaluronidases, and the effects of HA on angiogenesis are not clear. Specific HMW-HA molecules exhibit pro-angiogenic activity. For examples, Tang reported that local injection of HMW-HA measuring about 1630 kDa increases angiogenesis in a mouse model of hindlimb ischemia [10]. Galeano M reported that intravenous administration of HMW-HA (5000 kDa) improved wound healing in diabetic mice [11]. The HA content decreases in aging, and plays a significant role in wound healing.

In the present study, we used HMW-HA (1650 kDa), and investigated its effects on endothelial cells and angiogenesis. We further investigated the role of this HMW-HA in promoting wound healing in an old rat model by local injection.

## MATERIALS AND METHODS

### HMW-HA solution

HA powder (1650 kDa, Abcam, Shanghai, China. CAT#: ab143634, purity > 95%) was blended in the PBS at 4°C for 24 h to achieve total hydration (5 mg/mL), and stored at 4°C for usage.

### Endotoxin content determination

HA powder was opportunely dissolved in pyrogen free water. Limulus Amebocyte Lysate (LAL) test was performed to measure the amount of pyrogens (bacterial endotoxins) in the solution. ENDOSAFE^®^-PTS cartridge US License N.1197 by Charles River Endosafe was used. All operations were performed under conditions avoiding endotoxin contamination. Results were reported as endotoxin units (EU/mg) of HA powder.

### Cell proliferation assay

HUVECs were plated at a density of 1 × 10^4^ cells/well in complete medium containing 10% FBS in 96-well plates. After attachment of HUVECs, the medium was removed and replaced with complete medium containing 10% FBS either in the presence or absence of HMW-HA. Cells were cultured for 48 h, followed by the addition of 20 μL of methylthiazolyldiphenyl-tetrazolium bromide (MTT) reagent to each well, and the plates were incubated for 4 h at 37°C. The supernatants were discarded and 150 μL of DMSO was added to each well. The 96-well plate was oscillated at room temperature for 10 min. The wells without cells were used as the zero point of absorbance. The absorbance was measured using a microplate reader at 570 nm.

### Tube formation assay

Matrigel (200 µL) was polymerized in the wells of a 24-well plate at 37°C for 30 min. HUVECs (1 × 10^5^) were dispensed into each well in 1000 μL of complete medium containing 10% FBS with or without HMW-HA (1000 μg/mL). Cord morphogenesis of HUVECs was assessed using phase-contrast microscopy. Tubule structure was photographed 6 h after cell seeding under microscopy. Vascular cross points were counted in 5 randomly selected fields under microscopy in a blinded manner.

### *In vitro* assay of wound recovery

HUVECs were plated at a density of 4 × 10^5^ cells/well in complete medium containing 10% FBS in 6-well plates. After 90% confluence of cell growth, the cells were washed in PBS, and the cellular layer was wounded using a mechanical scarifier. The cells were rinsed again in PBS to remove loose and dislodged cells, and transferred into a fresh medium either in the presence or absence of HMW-HA (1000 μg/mL). Cells were cultured for 48 h. Movement of cells into the denuded area was quantified using a Seescan computerized image analysis system (Manchester, UK).

### Cellular apoptosis

First, after 48 h of endothelial cell culture under serum starvation with or without 1000 μg/mL, HMW-HA, Hoechst 33342 staining of cells was performed, and apoptotic cells were identified on the basis of morphological changes in nuclear assembly involving chromatin condensation and fragmentation. Annexin V and PI staining were used to detect apoptotic cells. In brief, after incubation with serum-free medium for 48 h, endothelial cells were digested with 0.25% trypsin and washed with PBS. After centrifugation at 2000 r/min for 5 min, cells were collected, followed by addition of 500 μL Binding Buffer, 5 μL Annexin V-FITC and 5 μL PI. Subsequently, they were mixed at room temperature in dark for 10 min, followed by flow cytometry within 1 h.

### Animals

Male Sprague-Dawley (SD) old rats aged 22 months and young rats aged 4 months were provided by the Experimental Animal Center of Jinzhou Medical University. All the experimental procedures were carried out in accordance with the recommended guidelines for the care and use of laboratory animals issued by the Chinese Council on Animal Research.

Rats were randomly and equally divided into two groups as follows: control (PBS) and treatment (HA). After inducing anesthesia via an intramuscular injection of 40 mg/kg ketamine and 4.0 mg/kg xylazine, the hair was shaved, and a 2.0 cm full-thickness wound was created on the dorsal surface of the left flank. Subcutaneously, 100 μL of 1000 μg/mL of HMW-HA was injected at five points around the edge of the wound, with 20 µL at each point. Furthermore, the effects of 1650 kDa HA on the wound healing in old rats were compared with the effects of different sized-HA from Sigma (HA2000-2400, HA300-500 and HA 1.2 kDa, Shang, China).

### HA quantification

HA quantification in the skin was performed as previously described [10]. In brief, the skin from young and old rats was removed and frozen in liquid nitrogen for HA determination. Frozen tissues (50 mg) were homogenized in 1.0 mL of 150 mM Tris–HCl, pH 8.3; 150 mM sodium chloride; 150 mM calcium chloride; 5 mM deferoxamine and 40 IU of protease K using a homogenizer. The sample was digested overnight at 55°C and the homogenates clarified by centrifugation at 4°C (18,000g for 5 min). Supernatants were collected and transferred to a boiling bath for 20 min to inactivate any proteases. The assay was carried out using a specific enzyme-linked protein assay test kit (cat no. 029-001, Corgenix, Cambridgeshire, UK) according to the manufacturer’s method.

### Measurement of blood flow

The measurement of blood flow was performed as previously described [9]. In brief, immediately after creating the wound in rats, blood flow in the wound area was measured using the Laser Doppler (PeriCam, PSI, Perimed). Subsequently, the blood flow was measured on days 7 and 14 during the wound healing.

### Capillary density

After 14 d, the rats were scarified. The skins around the wound were harvested, and embedded in paraffin. Cryosections measuring 4 μm in thickness were prepared. Endothelial cells were stained with monoclonal anti-CD31 primary antibody (BD Pharmingen, SD, CA, USA), followed by biotinylated anti-mouse IgG secondary antibody, and an avidin-HRP conjugate for color reaction (DAB paraffin IHC staining module, Ventana Medical Systems, Inc., Tucson, AZ, USA). Hemotoxilin was used for counter staining. The sections were analyzed by microscopy with five high-power fields randomly selected for each section. CD31-positive cells were counted in a blinded manner. The number of CD 31-positive cells in each field was used as an index of capillary density.

### Western blot

The HUVECs were washed twice with cold PBS and re-suspended in cold lysis buffer containing 20 mmol/L HEPES (pH 7.5), 150 mmol/L NaCl, 1.0 mmol/L ethylenediaminetetraacetic acid, 0.5% Triton X-100, and protease inhibitors (Roche). Similar quantities of total protein (20 μg) were separated by SDS-gel electrophoresis, transferred onto polyvinylidene fluoride membranes, and blocked overnight in blocking solution at 4.0 °C. To detect phosphorylation of AKT, Src and ERK, the membranes were incubated for 1.0 h with mouse monoclonal antibodies raised against human p-AKT (Cell Signaling Technology, Danvers, MA, USA), p-Src (Cell Signaling Technology, Danvers, MA, USA), and p-ERK (Cell Signaling Technology, Danvers, MA, USA), respectively, followed by anti-mouse or anti-rabbit secondary antibodies conjugated to horseradish peroxidase (Zymed, Inc., South San Francisco, CA, USA) for 1.0 h at room temperature. To detect the expression of HAS-2 and HAS-3 in the skin of aged rat, the wounded skin tissues were harvested at 7th day, and homogenized in lysis buffer. Similar quantities of total protein (40 μg) were separated by SDS-gel electrophoresis, transferred onto polyvinylidene fluoride membranes, and blocked overnight in blocking solution at 4.0°C. The membranes were incubated for 1.0 h with rabbit polyclonal antibodies raised against rat HAS-2 and rabbit monoclonal antibodies raised against rat HAS-3 (Abcam, Shanghai, China), respectively, followed by anti-rabbit secondary antibodies conjugated to horseradish peroxidase (Zymed, Inc., South San Francisco, CA, USA) for 1.0 h at room temperature. The ECL Plus system (Amersham Biosciences UK, Little Chalfont, UK) was used to reveal the signals.

### Statistical analyses

Data are presented as the mean ± standard deviation (SD). Statistical comparisons were performed using analysis of variance followed by Bonferroni/Dunn tests. *P* < 0.05 was considered statistically significant.

## RESULTS

### Endotoxin amount in HA samples

In order to exclude the effects by endotoxin in HA, we detected endotoxin levels in all the HA samples used. The results showed the endotoxin amount for all hyaluronan powders was less than 0.01 EU/mg. This data proved that LPS and/or endotoxin are below a guard level and therefore cellular phenomenon should be driven by hyaluronan rather than impurities on the tested sample alone.

### HMW-HA promotes the proliferation of HUVECs

**Figure 1:**
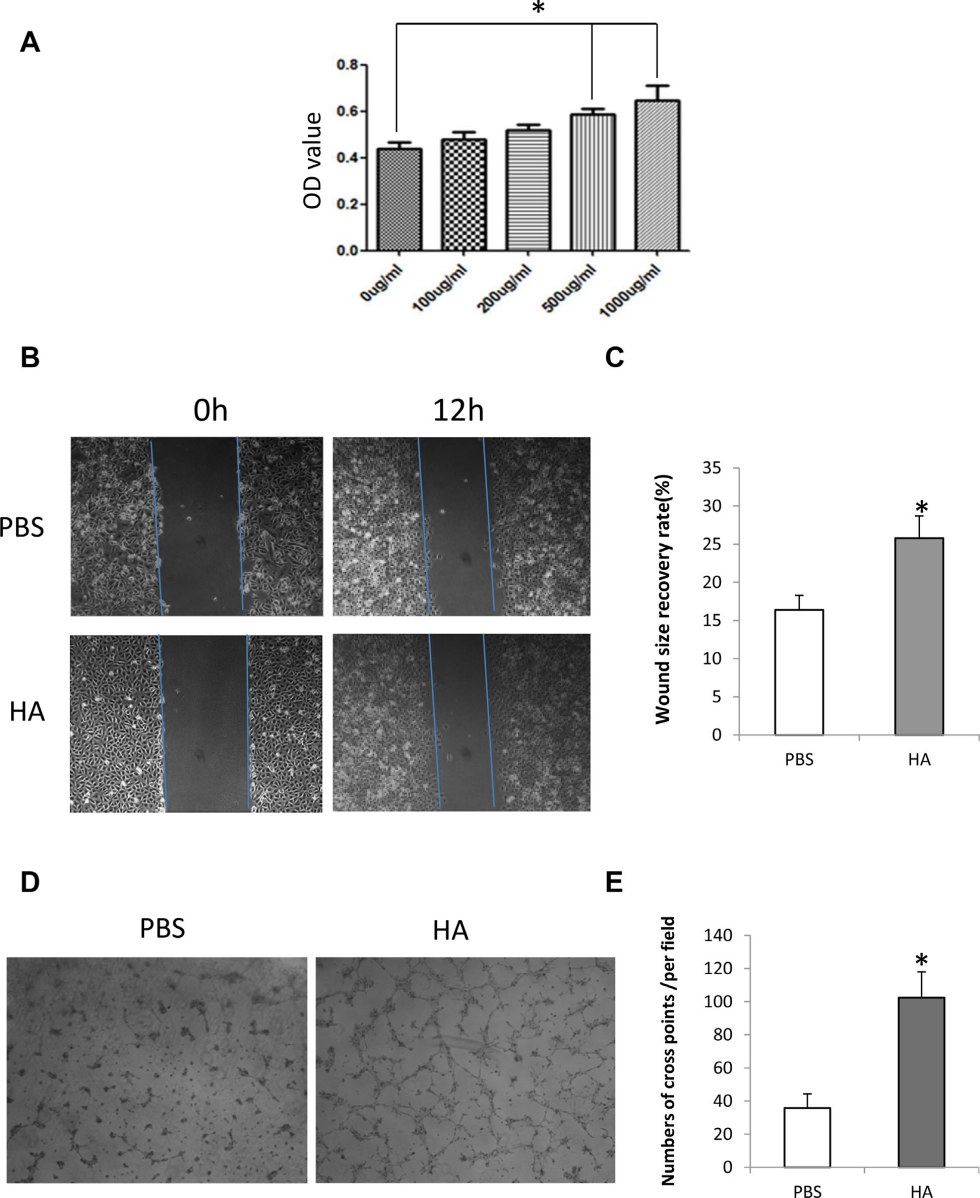
HMW-HA promotes proliferation, migration and tube formation of endothelial cells *in vitro* (**A**) Effect of HMW-HA on the proliferation of endothelial cells. MTT assay shows that HMW-HA at concentrations of 500 and 1000 μg/mL significantly increase the proliferation of endothelial cells after 48 h of cell culture (*p* < 0.05). (**B**) Effect of HMW-HA at a concentration of 1000 μg/mL on the migration of endothelial cells: upper panel, PBS-treated group; lower panel, HMW-HA-treated group. (**C**) HMW-HA significantly improves the migration of endothelial cells after 12 h of cell culture in endothelial cells (*p* < 0.05). (**D**) Effect of HMW-HA at a concentration of 1000 μg/mL on endothelial cell tube formation: left panel, PBS-treated group; right panel, HMW-HA-treated group. (**E**) HMW-HA significantly increases tube formation after 6 h of cell culture in Matrigel (*p* < 0.01).

To determine the effect of HMW-HA on the proliferation of HUVECs, an MTT assay was performed. The concentration of HMW-HA ranged from 0 to 1000 μg/mL. The results showed that after 48 h of treatment, endothelial cell proliferation increased more significantly at the concentration of 500 and 1000 μg/mL compared with the control (*p* < 0.05, Figure 1A).

### HMW-HA promotes wound healing *in vitro*

An *in vitro* wound recovery assay was used to determine the effect of HMW-HA on the migration of endothelial cells. After creating a wound on the sample plate, 1000 μg/mL HMW-HA was added to the culture medium. The results showed that 48 h later, the wound recovered more significantly than the control (*p* < 0.01, Figure 1B and 1C).

### HMW-HA promotes tube formation of endothelial cells

To determine the effect of HMW-HA on angiogenesis *in vitro*, the tube formation of endothelial cells was assessed. HMW-HA (1000 μg/mL) was added to the culture medium. After 6 h of culturing endothelial cells in Matrigel, endothelial cell tubes were formed more significantly than in the control (*p* < 0.01, Figure 1D and 1E).

### HMW-HA decreases apoptosis of endothelial cells

**Figure 2:**
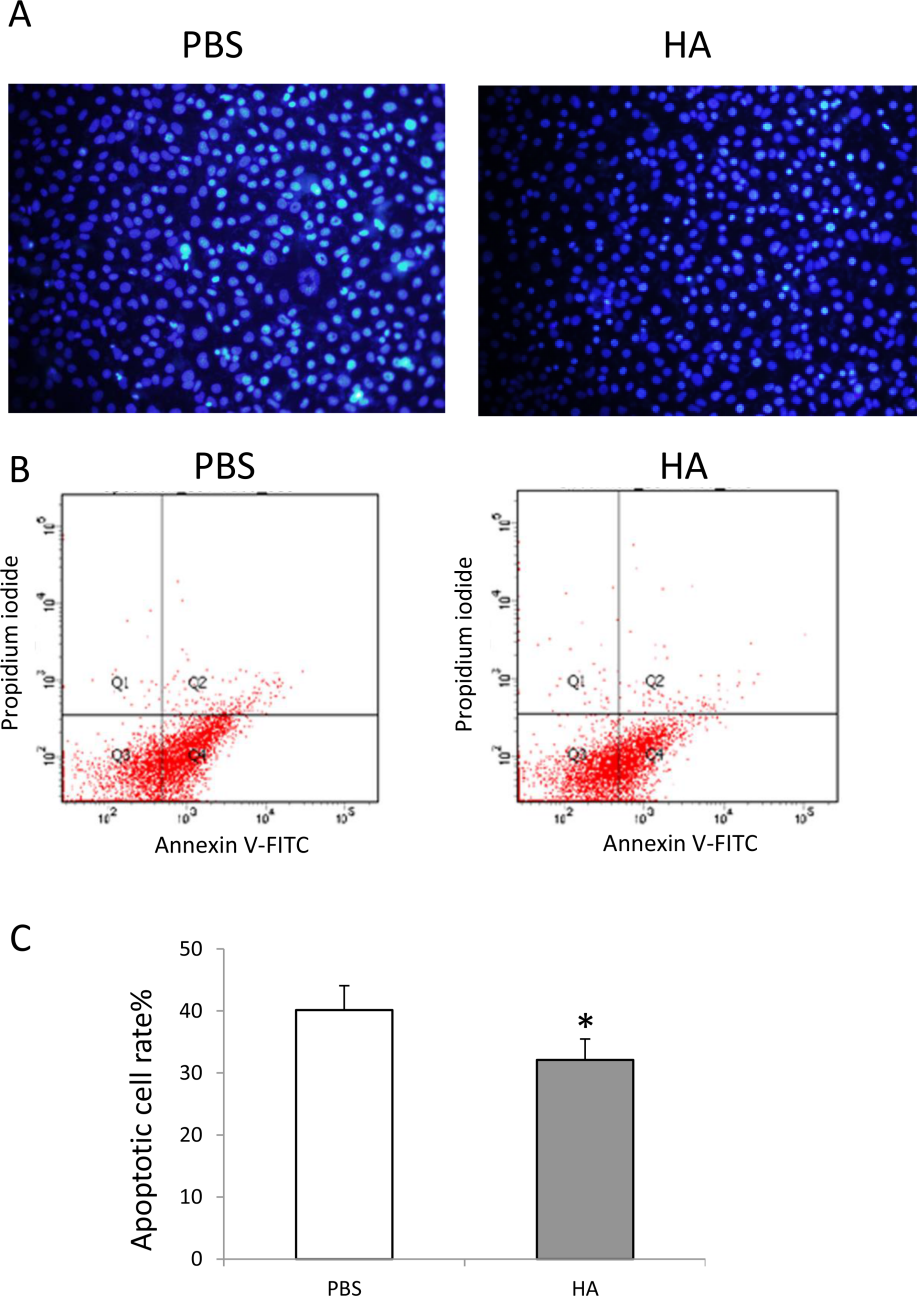
HMW-HA decreases apoptosis of endothelial cells (**A**) Hochest 33342 staining of endothelial cells cultured with serum starvation for 48 h; (**B**) cytometery analysis of apoptosis of endothelial cells cultured with serum starvation for 48 h; (**C**) HMW-HA treatment significantly decreases apoptosis of endothelial cells compared with PBS treatment (*p* < 0.01).

To determine the effect on the survival of endothelial cells cultured with serum-free medium, HMW-HA (1000 μg/mL) was added to the culture medium. After 48 h of treatment, Hoechst 33342 staining was performed. The results showed that HMW-HA obviously decreased apoptosis of endothelial cells (Figure 2A). Annexin V and PI staining was also performed and analyzed using flow cytometry. The results showed that HMW-HA significantly decreased apoptosis of endothelial cells (*p* < 0.01, Figure 2B and 2C).

### HMW-HA increases the expression of p-Src, p-ERK and p-AKT

**Figure 3:**
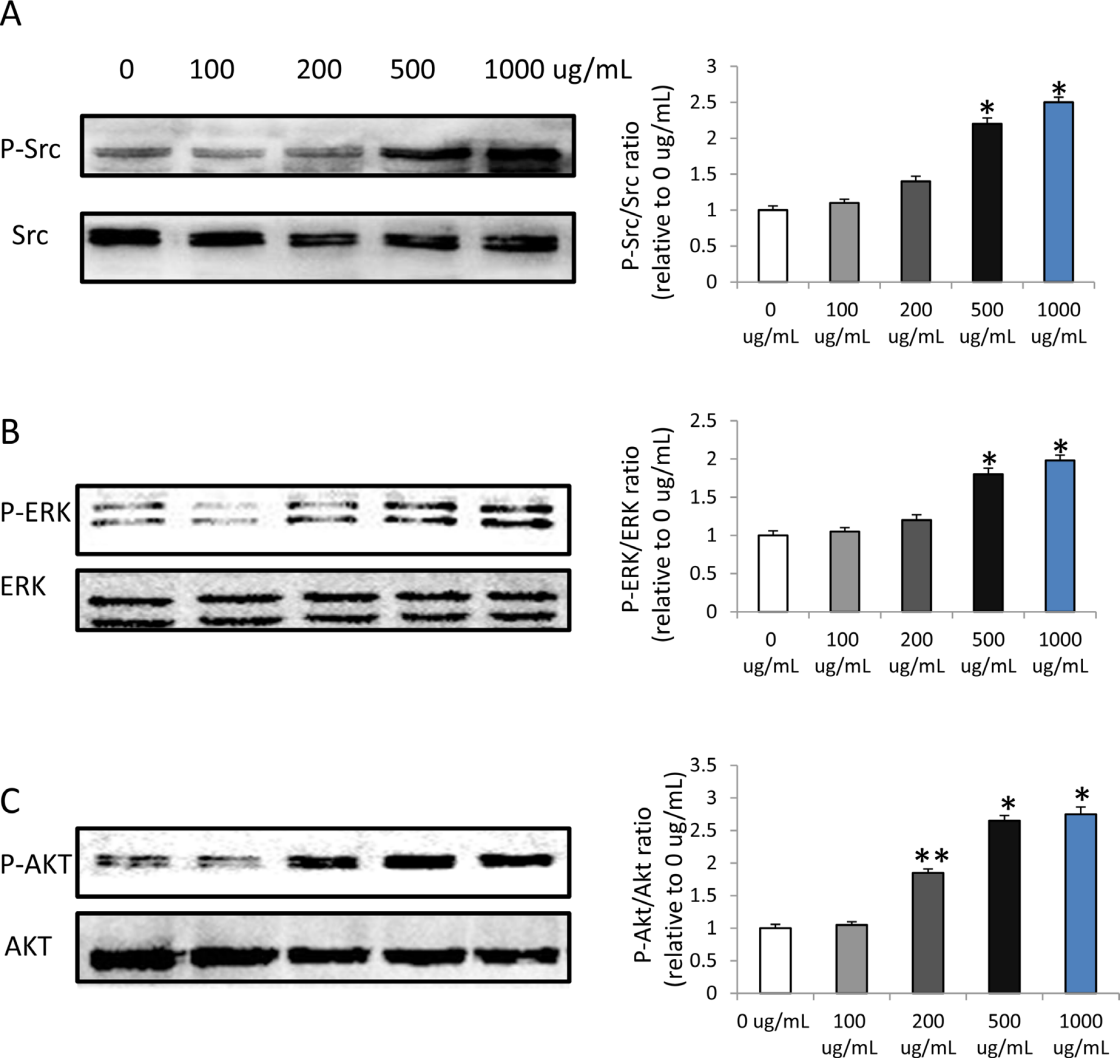
HMW-HA increases the phosphorylation of Src, ERK and AKT in endothelial cells by western blot detection (**A**) HMW-HA increases the phosphorylation of Src at concentration of 500 and 1000μg/mL, (^*^p < 0.01 versus 0 μg/mL HMW-HA), *n* = 3 in each group. (**B**) HMW-HA increases the phosphorylation of ERK at concentration of 500 and 1000μg/mL, (^*^p < 0.01 versus 0 μg/mL HMW-HA), *n* = 3 in each group. (**C**) HMW-HA increases the phosphorylation of AKT at concentration of 200, 500 and 1000μg/mL, (^*^p < 0.01 versus 0 μg/mL HMW-HA; ^**^p < 0.05 versus 0 μg/mL HMW-HA), *n* = 3 in each group.

To determine the mechanisms underlying angiogenesis following HMW-HAtreatment, we focused on the downstream signaling pathways of MARK and AKT. The results showed that HMW-HA increased the phosphorylation of Src, ERK and AKT in endothelial cells (Figure 3A, 3B and 3C), suggesting increased angiogenesis by promoting proliferation, migration and survival of endothelial cells.

### Skin content of hyaluronan is decreased; expression of HAS-2 and HAS-3 in wounded skin tissues decreased, and wound healing delayed in old rats

**Figure 4:**
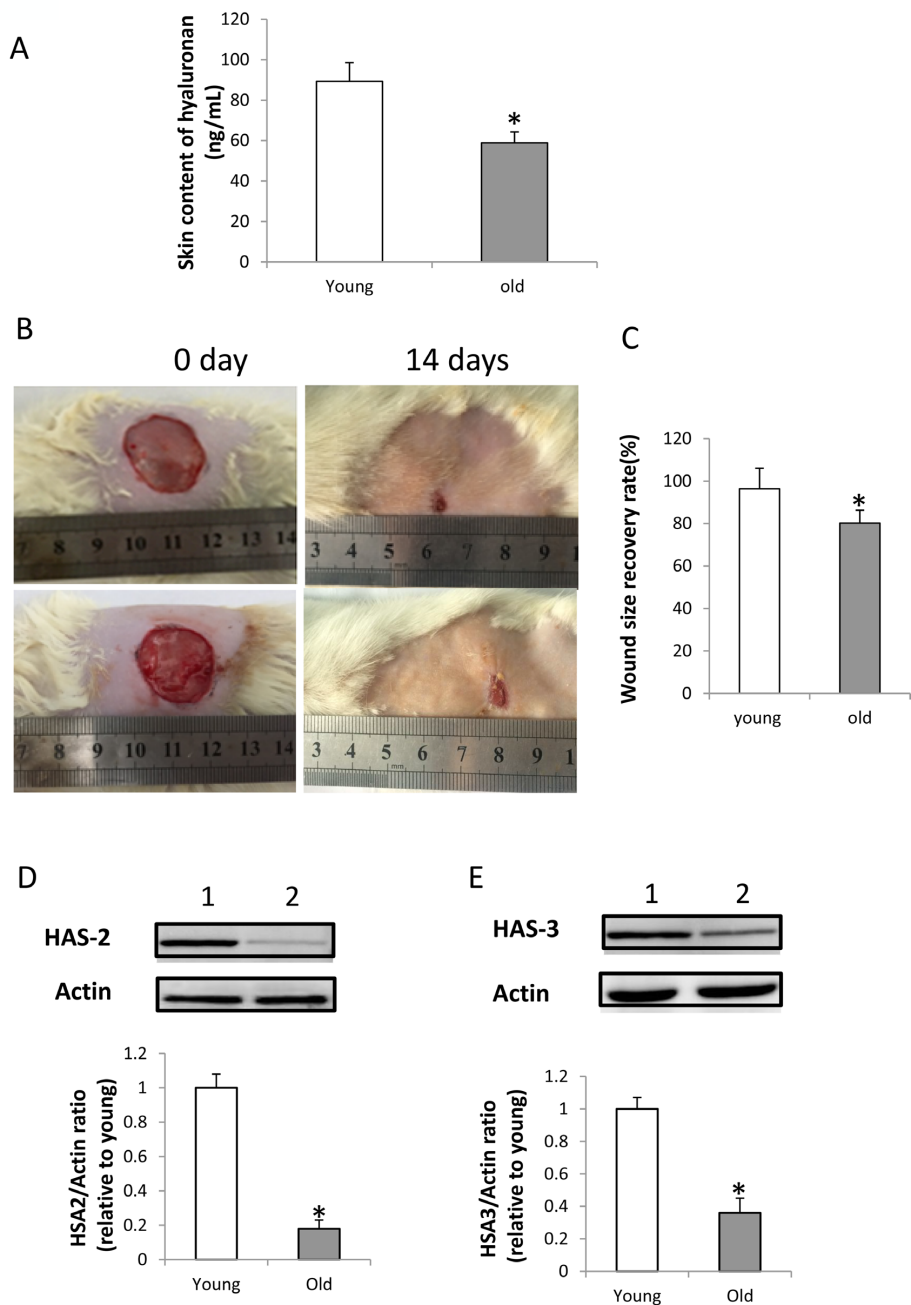
HA content, expression of HAS-2 and HAS-3 in the wounded skin and wound healing in old rats (**A**) HA content in young and old rats. HA content in the skin of old rats was significantly less than in young rats (*n* = 5, *p* < 0.01); (**B**) Wound healing in young and old rats: upper panel, wound healing in young rat; lower panel, wound healing in old rat. (**C**) Wound healing is significantly delayed in old rats compared with young rats (*n* = 5, *p* < 0.05). (**D**) Expression of HAS-2 was decreased in the wounded skin of old rats at 7th day by western blot detection. Lane 1, skin of young rats; lane 2, skin of old rats. The Expression of HAS-2 in the wounded skin of old rats were significantly decreased compared with that of young rats (*n* = 3, *p* < 0.05). (**E**) Expression of HAS-3 was decreased in the wounded skin of old rats at 7th day by western blot detection. Lane 1, skin of young rats; lane 2, skin of old rats. The Expression of HAS-3 in the wounded skin of old rats was significantly decreased compared with that of young rats (*n* = 3, *p* < 0.05).

A specific enzyme-linked binding protein assay was performed to detect the content of intact hyaluronan in young and old rats. The mean hyaluronan level in the skin ranged from 68.7 ± 5.3 ng/mL in young rats to 43.8 ± 4.5 ng/mL in old rats. It suggested a marked decrease in hyaluronan content of aging rats (*p* < 0.01, Figure 4A). Furthermore, the wound healing model showed that the wound healing is significantly delayed in old rats than that in young rats (*p* < 0.05, Figure 4B and 4C), and the expression of HAS-2 and HAS-3 in wounded skin of old rats at 7th day was decreased (Figure 4D and 4F).

### Local injection of HMW-HA promotes wound healing in old rats

**Figure 5:**
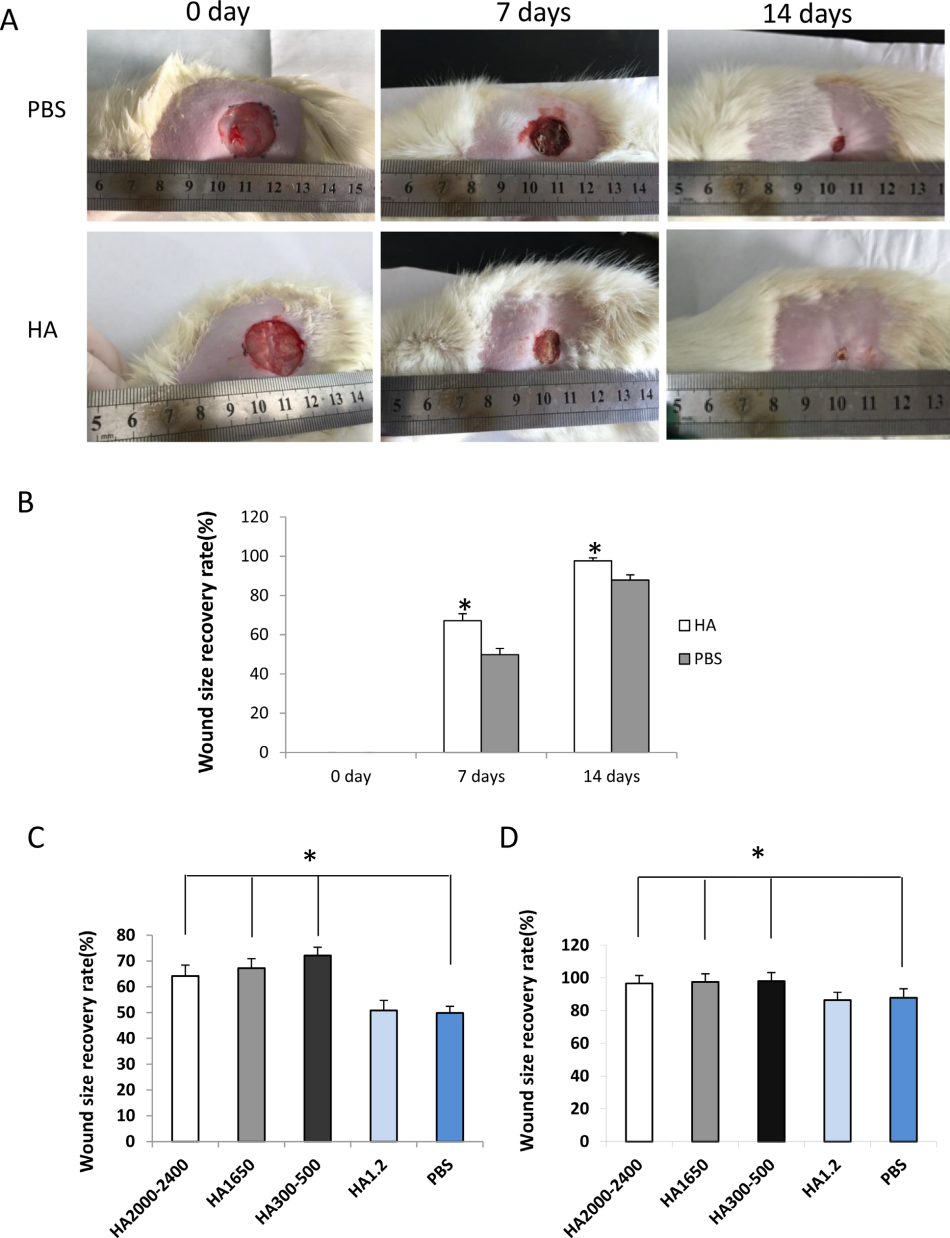
Local injection of HMW-HA decreases wound size in old rats (**A**) Upper panel, wound treated with PBS; lower panel, wound treated with HMW-HA; (**B**) HMW-HA significantly decreases wound size in old rats compared with the controls (*n* = 5, *p* < 0.05). (**C**) HA2000-2400, HA1650 and HA300-500 promoted wound healing in old rats at 7th day, however, HA1.2 did not promoted wound healing in old rats (*n* = 5, *p* < 0.05). (**D**) HA2000-2400, HA1650 and HA300-500 promoted wound healing in old rats at 14th day, however, HA1.2 did not promoted wound healing in old rats (*n* = 5, *p* < 0.05).

To determine the effect of HMW-HA on wound healing *in vivo* in aging, the old SD rats were used to develop the wound model. Our data showed that 1000 μg/mL HMW-HA was effective in promoting proliferation, migration and tube formation of endothelial cells *in vitro.* Therefore, 1000 μg/mL HMW-HA was locally injected into the subcutaneous site around the wound. The wound size was measured on days 0, 7 and 14 during the wound recovery process. The results showed that local injection of 1000 μg/mL HMW-HA significantly decreased wound size (*p* < 0.05, Figure 5A and 5B). We also performed the experiments to compare the effects of different sized-HA on the wound healing in old rats. The results showed that HA2000-2400, HA1650 and HA300-500 significantly promoted wound healing in old rats (*p* < 0.01, Figure 5C and 5D), however, HA1.2 did not promoted wound healing in old rats.

### HMW-HA improves blood flow

**Figure 6:**
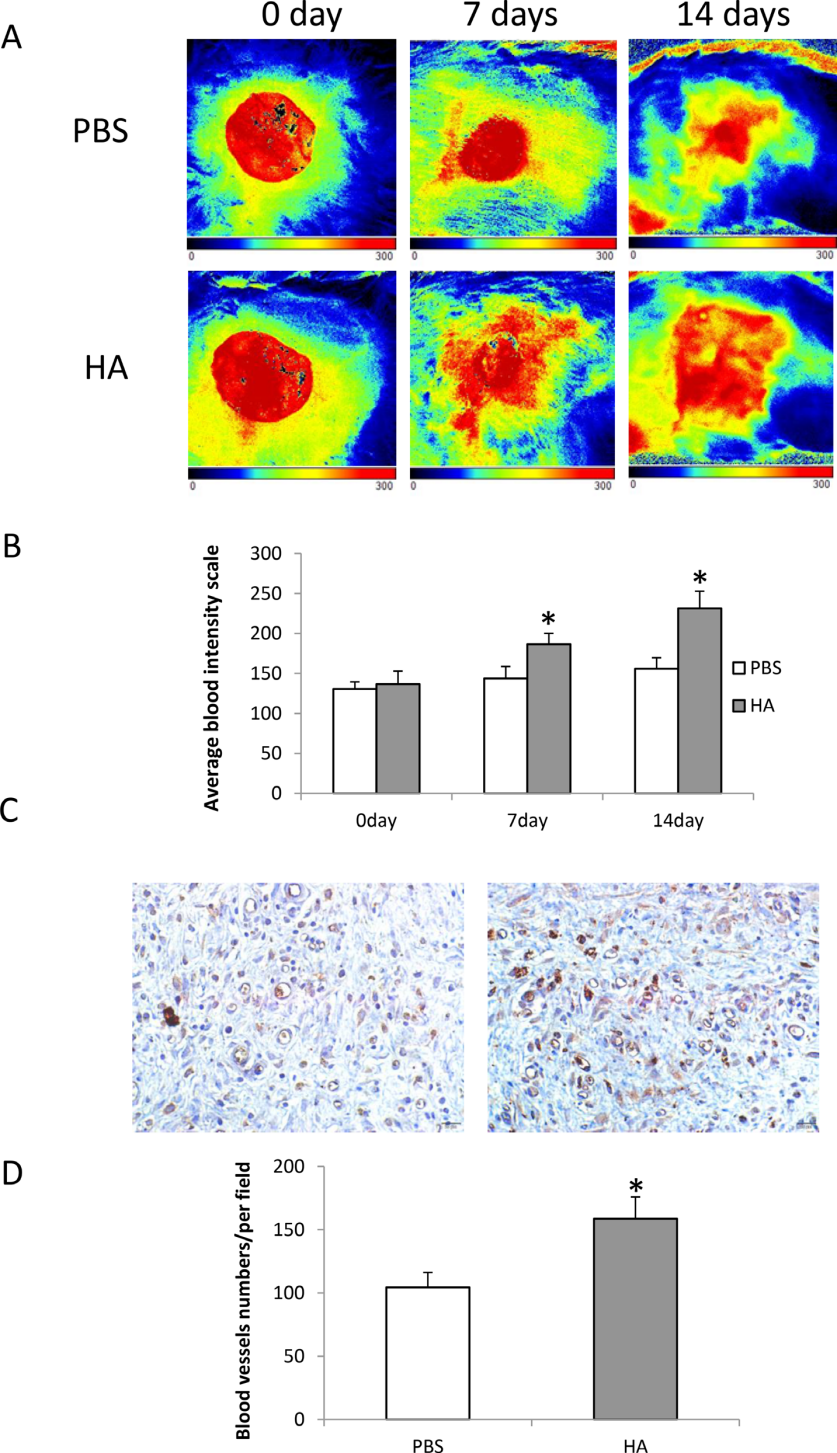
Local injection of HMW-HA increases blood flow in the wounded area of old rats (**A**) Laser Doppler imaging of blood flow in the wound area: upper panel, wound treated with PBS; lower panel, wound treated with HMW-HA; (**B**) HMW-HA significantly increases blood flow in the wound area of rats compared with the controls (*n* = 5, *p* < 0.01); (**C**) CD31 immunostaining in wound area: left panel, tissue from the wound treated with PBS; right panel, tissue from the wound treated with HMW-HA (Bar = 20 μm); (**D**) HMW-HA significantly increases capillary density in the wound area of rats compared with the controls (*n* = 5, *p* < 0.01).

Blood flow around the wound area was measured using laser Doppler on days 0, 7, and 14 during the wound recovery process. The average blood flow–intensity was compared between the groups treated with HMW-HA and PBS. The results showed that local injection of 1000 μg/mL HMW-HA improved blood supply to the wound area more significantly compared with that of the control (*p* < 0.05, Figure 6A and 6B).

### HMW-HA increases capillary density

To determine the effects of HMW-HA on angiogenesis *in vivo*, the skin tissues around the wound area of old rats were harvested on day 14 and immunohistochemistry was performed to detect CD31 expression. The results showed that local injection of 1000 µg/mL HMW-HA significantly increased the capillary density in the wound area (*p* < 0.01, Figure 6C and 6D).

## DISCUSSION

It is generally accepted that defective angiogenesis contributes to delayed wound healing in aging. The decline in neovascularization has been attributed to decreased proliferation and migration of endothelial cells in the wound bed. Although several studies investigated the role of HMW-HA and angiogenesis [10, 11], little is known about its effects on angiogenesis in elderly patients. In this study, we investigated the effects of local injection of HMW-HA (1650 kDa) on wound healing in old rats, and found that it promoted wound healing by increasing angiogenesis. HA is a natural product and contains no protein. Therefore, there are no concerns about potential health risks that are associated with other methods of treatment such as gene therapy.

HA within the epidermis displays rapid turnover [12, 13]. HA catabolism in the epidermis is closely correlated with its synthesis and degradation. Under physiological conditions, HA is synthesized by several HA synthases (HAS) [14], and HA fragments of low molecular mass are produced by hyaluronidases or oxidation [15]. Under pathologic conditions, such as injury, the production of HA is enhanced [16]. It interacts with the surrounding cells triggering a series of pathological changes in the wound, including angiogenesis [17]. However, an age-related decrease in HA production in skin has been detected in both mice [12] and humans [13]. Our study showed that the HA is decreased in the skin of old rats, and the expression of HAS-2 and HAS-3 in wounded skin of old rats is decreased. It is well known that aging is associated with impaired wound healing, which is consistent with low levels of HA deposition and HA size modifications in skin aging and impaired wound healing. Thus, HA supplementation led to beneficial effects on wound healing in aging. We found that local injection of HMW-HA significantly promoted wound healing in old rats, indicating potential therapeutic role clinically. Interestingly, we also found HA (300-500 kDa) promoted the wound healing in old rats. This indicated that different sized-HA may have different roles in the wound healing in aging. It has been reported that HA (1500 kDa) provided benefits to endothelial cell sustenance, proliferation and normal functionality [18]. In this study, we clearly proved that HMW-HA (1650 kDa) promoted wound healing through increasing angiogenesis. However, D’Agostino A et al reported that HAs (500, 1400 and 1800 kDa) promoted the wound reparation process by the effects on keratinocytes [19]. We think this is one of the reasons that may explain why both HMW-HA (1650 kDa) and medium-sized HA (300-500 kDa) promoted wound healing in our *in vivo* experiment. Further studies are needed to study the small to intermediate sized HA in the wound healing in aging.

HA and its cell surface receptors play pivotal roles in cell proliferation, migration and invasion. CD44 and RHAMM are the two main HA receptors in vascular endothelial cells of human. Both CD44 and RHAMM are the targets for transduction of HA-induced angiogenesis [20–22]. These two receptors work in tandem to facilitate neoangiogenesis [23]. HA induces rapid CD44-dependent activation of multiple isoforms of PKC, Raf-1 kinase, MEK-1, and ERK1/2, resulting in proliferation of endothelial cells [20]. HA also binds to the RHAMM receptor and induces tyrosine phosphorylation of p125FAK, paxillin, p42/44, and extracellular ERK1/2, resulting in cell proliferation [22]. Furthermore, the interaction of HA-CD44 with RHAMM influences physiological and cellular functions. Cell surface RHAMM interacts with CD44, HA and growth factor receptors to activate protein tyrosine kinase signaling cascades that activate the RRK1/2/MAP kinase cascade in a c-Src/MeRK-1//ERK1/2 dependent manner [24]. Recently, Galeano M et al demonstrated that systemic administration of HMW-HA stimulates angiogenesis in diabetic mice by increasing the expression of TGF beta 1 [11] suggesting that HA application also influenced angiogenesis-related cytokine production in the wound. In this study, we found that HMW-HA increases phosphorylation of Src, ERK and AKT in endothelial cells. Signaling by HMW-HA increases angiogenesis via proliferation and migration of endothelial cells, and protection against apoptosis.

In summary, HMW-HA increases endothelial cell proliferation, migration and tube formation, and protects endothelial cells against apoptosis. Local injection of HMW-HA promotes wound healing in old rats by increasing angiogenesis. The mechanisms include increased phosphorylation of Src, ERK and AKT. Our study provides a new method of wound healing in elderly patients in clinical practice.
